# Local Experimental Intracerebral Hemorrhage in Rats

**DOI:** 10.3390/biomedicines9060585

**Published:** 2021-05-21

**Authors:** Ekaterina Vasilevskaya, Aleksandr Makarenko, Galina Tolmacheva, Irina Chernukha, Anastasiya Kibitkina, Liliya Fedulova

**Affiliations:** V.M. Gorbatov Federal Research Center for Food Systems, Russian Academy of Sciences, 109316 Moscow, Russia; vivarium@vniimp.ru (A.M.); tgs2991@yandex.ru (G.T.); imcher@inbox.ru (I.C.); anastasjya@list.ru (A.K.); fedulova@vniimp.ru (L.F.)

**Keywords:** biomodel, stroke, hemorrhage, neurology, rats, behavior

## Abstract

(1) Background: Hemorrhagic stroke is a lethal disease, accounting for 15% of all stroke cases. However, there are very few models of stroke with a hemorrhagic etiology. Research work is devoted to studying the development of cerebrovascular disorders in rats with an intracerebral hematoma model. The aim of this study was to conduct a comprehensive short-term study, including neurological tests, biochemical blood tests, and histomorphological studies of brain structures. (2) Methods: The model was reproduced surgically by traumatizing the brain in the capsula interna area and then injecting autologous blood. Neurological deficit was assessed according to the McGrow stroke-index scale, motor activity, orientation–exploratory behavior, emotionality, and motor functions. On Day 15, after the operation, hematological and biochemical blood tests as well as histological studies of the brain were performed. (3) Results: The overall lethality of the model was 43.7%. Acute intracerebral hematoma in rats causes marked disorders of motor activity and functional impairment, as well as inflammatory processes in the nervous tissue, which persist for at least 14 days. (4) Conclusions: This model reflects the situation observed in the clinic and reproduces the main diagnostic criteria for acute disorders of cerebral circulation.

## 1. Introduction

Acute and chronic disorders of cerebral circulation are problems that affect many people. To develop adequate methods for the prevention of various stroke subtypes and rehabilitation after the disease, it is necessary to conduct comprehensive research, including using animal biomodels [1,2]. The success of such studies depends on the choice of an experimental model since an inadequate model can lead to limitations that compromise the results and analysis. In addition, extrapolating the results of animal models to humans can be unreliable. Rodents are mainly used for stroke modelling due to cost considerations, ethical considerations, the availability of standardized neurobehavioral assessments, and the simplicity of the physiological monitoring [3,4,5].

Modern publications mention two main types of models for higher nervous activity disorders: the use of chemical agents for post-stroke syndrome development in animals and surgical models of spontaneous stroke [6]. Both mice and rats are used as research objects. However, due to the larger size of the latter (in adulthood, rats weigh about 8–10 times more than mice), experiments with rats guarantee several practical advantages, especially concerning surgical procedures. Models of the brain and spinal cord injury using rats have a great translational value [7]. In addition, rats and mice showed significant differences in the plasticity of their hippocampal and cortical neurons [8]. It was found that the rate of neurogenesis in the hippocampus of sexually mature rats is much higher than in mice and, importantly, in rats, new cells mature about 2 weeks earlier than in mice; in addition, the probability of their activation is ten times higher [9].

There also are rat strains susceptible to stroke (spontaneously hypertensive SHR and stroke-prone spontaneously hypertensive SHRSP rats) that can be used as an experimental model, characterized by a high frequency of spontaneous strokes as well as increased sensitivity to experimentally induced focal cerebral ischemia [10]. The advantages of using SHR rats in stroke research are concomitant hypertension presence and the development of reproducible infarction of adequate size after distal middle cerebral artery occlusion. At the same time, this model has a significant disadvantage—SHR and SHRSP rats are expensive, and a high mortality of the animals is observed at a certain age; in addition, these rats are resistant to therapy [11]. Furthermore, SHRSP rats usually have ischemic, or less frequently, hemorrhagic stroke in the cortex, rather than in the brainstem, cerebellum, or basal ganglia, as in patients with hypertension [12].

It is worth noting the relatively small number of stroke models with a hemorrhagic etiology, this while hemorrhagic stroke accounts for approximately 15% of all stroke cases and leads to high mortality. The main models were developed in the twentieth century and are based on direct injection of autologous blood or bacterial collagenase into various brain regions (autologous blood injection model and bacterial collagenase injection model) [3,13,14,15,16]. However, they are most often used for large animals (primates, pigs, and rabbits) and less often for rats and mice [17,18,19]. One of the disadvantages of the autologous blood injection model is the use of an anticoagulant agent, which leads to additional internal injuries and retrograde penetration of heparinized blood through the reverse channel, as well as into non-target areas of the brain [18]. The method developed earlier [20] for reproducing experimental acute intracerebral hemorrhage allows us to recreate limited damage to brain structures localized in the capsula interna area, which is the most reliable and close to the human model, but not all aspects of this model have been considered.

Acute stroke period assessment is a critical stage used to understand stroke severity, treatment options, and prognosis, and it serves as the main tool for identification biomarkers, but the recovery period and its characteristics are also of great importance [21]. To better analyze the pathophysiology of the acute and recovery periods in the hemorrhagic stroke model according to [20], for adequate in vivo studies, a comprehensive short-term study was conducted, including neurological tests, biochemical blood analysis, and histomorphological studies of brain structures.

## 2. Materials and Methods

### 2.1. Animals

The study was carried out on male Wistar rats (*n* = 30), 10 weeks old, and weighing 240 ± 25 g, obtained from the Federal State Budgetary Institution of Science “Scientific Center for Biomedical Technologies of the Federal Medical And Biological Agency”, Andreevka Branch (Moscow Region, Solnechnogorsk District, Andreevka Settlement).

Animals were acclimatized to handling and the new facilities in harmonious groups for seven days prior to the experimental work. Visual inspection of the animals showed a normal health status over this time. A normal health status was confirmed by visual inspection of the animals and daily weighting during acclimatization. Animals were provided with cage substrate (Lignocel BK 8–15/LIGNOCEL, J. Rettenmaier & Sohne GMBH+CO KG, Sulzbach-Rosenberg, Germany) and a plastic shelter (Tecniplast, Milan, Italy). In the 3 days before surgery, animals were provided with nesting material for nest assembly. The cages were changed once every 6–7 days (but not less than three days before behavioural testing).

No supplemental diet was used in this experiment; after surgery, the rats’ diet (ad libitum) consisted of a complete feed compound (Laboratorkorm, Moscow, Russia).

Rats were kept in T-IV polycarbonate cages (Tecniplast, Milan, Italy), in groups of 3–4 individuals. The animals were kept under controlled environmental conditions: air temperature 21 ± 2 °C and relative humidity 50–60%. Lighting in the experimental rooms was artificial (12 h/day, 12 h/night), and daylight was inverted (light period from 6 p.m. to 6 a.m.); behavioral tests were performed in the dark period under dim red-lighting conditions, because rats are more active during the dark period [22]. 

### 2.2. Animal Model

To study the cerebrovascular disorders’ development, the rats were divided into the following groups:

1. Intact—intact animals kept under equal conditions (*n* = 6).
2. Sham—sham-operated animals (*n* = 8) underwent anesthesia and surgery with craniotomy without brain traumatization.
3. Model—operated on animals with reproduced intracerebral hematoma (*n* = 16)

Surgical operations were accompanied by the appropriate veterinary and medical procedures for preoperative preparation of the animals and postoperative care [23]. All non-sterile materials potentially in contact with the operating field (instruments, suture material, bandages, napkins, cotton wool, etc.) were sterilized with hot steam (121 °C, 20 min). The equipment and materials used in the operation (surgical table, stereotaxis, etc.) were treated with a disinfectant solution (0.5% solution of chlorhexidine biglucanate in 70% ethyl alcohol), followed by rinsing with sterile water.

Before the operation, the animals were subjected to food deprivation for 6 h, without restricting access to water. Fasting prior to surgery was used as preconditions against a variety of complications in preclinical models [24].

Immediately before the operation, the animal was anesthetized for 40 min with a mixture of Xila (Interchemie werken, De Adelaar, B.V, Castenray, the Netherlands) in a 10 mg/kg dose and Zoletil 100 (Virbac, Carros, France) in a 20 mg/kg dose intramuscularly [25].

Then, the animals were placed in an empty cage with a paper towel pad and closely monitored until anesthesia occurs. Anesthesia was verified by the disappearance of the reaction to pain stimuli (prick of the paw) and inhibition of the corneal reflex.

During general anesthesia and in the immediate post-operative period, the animal’s body temperature was maintained by insulation with rectal temperature control (VET1-R, Parthner-Agro SPB, Sankt-Peterburg, Russia). To warm the animals during and after the operation, we used a heating device for animals (Zoomed HM-1, Zoomed, Zelenograd, Russia) with a circulating-warm-water blanket (40 × 60 cm), with a feedback heating system that cuts out when a normal body temperature is reached; the heating temperature was 38.5 °C.

Pulse oximetry (Utech UT100, Utech, Shanghai, China) was used to ensure physiological stability under anesthesia, registering the tissue oxygen saturation and heart rate with a sensor placed on the tail.

Each animal was fixed with its back upon a surgical table and tied with loop-like knots behind its paws. To prevent the cornea from drying out, an eye gel Oftagel (Santen OY, Osaka, Finland) was dripped into the eyes. In the places of planned incisions, fur was removed, and the surgical field was treated from the center to the periphery with sterile swabs moistened with disinfectant. After treatment, the operating field was isolated with sterile wipes. 

**Figure 1:**
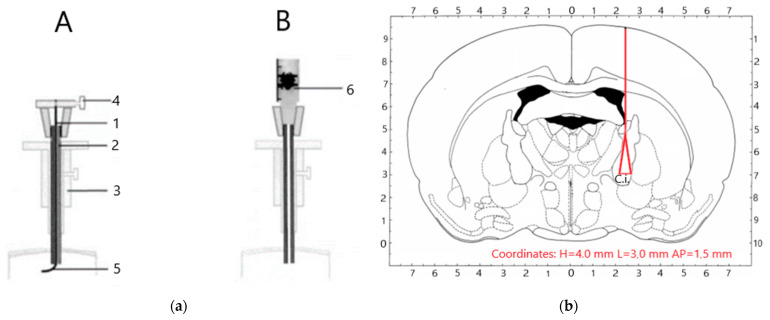
Scheme of the procedure of acute hemorrhagic stroke in the capsula interna: (**a**) Mandrel device: A—device is in position with extended mandrel; B—device after injection in the structure of the brain: 1—tungsten stylet-knife; 2—guiding needle cannula; 3—holder of the stereotaxic device; 4—latch top portion mandrel-knife; 5—cutting the bottom end mandrel-knife [20]. (**b**) Stereoscopic model [27] for simulating acute hemorrhagic stroke in the internal capsule. The red line and the red triangular area correspond to the post-surgery location of the damage. C.i.—Capsula Interna.

During the operation, a sagittal dissection of the integumentary tissues in the frontal, parietal, and occipital regions was performed; the periosteum was removed from the bones of the cranial vault. Modelling of unilateral stroke was carried out according to [20,26]. Under the technique, holes were drilled in the projection of the inner capsule using the stereotaxis device RWD 68025 and microdrill RWD 78001 (RWD Life Science Co., LTD, Shenzhen, Guangdong, China), with coordinates H = 4.0 mm, L = 3.0 mm, and AP = 1.5 mm from bregma according to [27] in the area of the right hemispheres; the diameter of the trepanation hole was 2 mm. Then the dura mater was pierced using a sharpened cannula needle (diameter 0.8 mm) with a rubber retainer and immersed to the required depth (4 mm). Brain traumatization in the inner capsule was carried out with a mandrel, using clockwise rotational movements (5–6 times), resulting in “cone-shaped” undercutting of the brain tissue and damage to the vessels in the area of the capsula interna (Figure 1).

An additional 100 μL of autologous blood was injected into the indicated area 2–3 min after trauma with a syringe (the blood was taken from the tail vein). Undercutting the vessels with a mandrel and introducing autologous blood into the site of injury is necessary to standardize the morphology, size, and location of the blood deposits [27]. The next incision was sutured with an intermittent suture with needles triangular in cross-section, and the suture was treated with an alcoholic solution of brilliant green. 

In sham-operated animals, 5 mm-long skin incisions were made, holes were drilled in the skull, and stitches were applied as in group 3 rats.

After surgery, each animal was placed in a postoperative box until it was completely out of anesthesia. 

Inter-animal housing, feeding, and handling practices before and after stroke was ensured. Animals were returned to the same group of animals they were with before surgery within 24 h after stroke. Each cage contained 2 sham-operated and 2 stroke animals, because social housing of rats with a healthy (non-stroke) partner immediately after stroke reduces mortality compared to housing with a stroke partner. Post-surgery care regarding bedding included cage substrate (Lignocel BK 8–15/LIGNOCEL, J. Rettenmaier & Sohne GMBH + CO KG, Sulzbach-Rosenberg, Germany) and nests.

Animals were monitored at least 6 times a day during the first 72 h post-stroke; the sensorimotor deficit and motor skills, water and food consumption, weight, and presence/absence of urine/feces were monitored, so too the surgical wound, breathing, and handling behavior were assessed [28].

To avoid dehydration, 1–2 mL/100 g of warm (35–37 °C) sterile Ringer’s solution was administered subcutaneously after the operation. Loose pellets were supplied for seven days post-stroke on the cage floor.

Further postoperative care included daily monitoring of the animal conditions: post-surgery pain in rats was assessed observing the following: decreased activity, piloerection, an ungroomed appearance, self-mutilation, abnormal stance or a hunched posture, respiration rapid and shallow with grunting or chattering on expiration, dilated pupils, porphyrin secretion (“red tears”) around the eyes and nose, vocalization and unusually aggressive when handled, changes in feeding activity, and group behavior or grooming [29,30,31].

If signs of pain and distress were detected [26], Xylazine in a 5–12 mg/kg dose was administered subcutaneously every 2–4 h [32] for the1st and 2nd days post-surgery for all animals, and special attention was paid to the condition of the sutures. 

Animal body weights were measured daily using an electronic laboratory balance (Adventurer Pro AV 2101, OHAUS, Parsippany, NJ, USA).

Humane endpoint criteria were selected [28,30,33]:

(a) Animal not moving, unresponsive to stimulation, or in a lateral recumbent position.
(b) Weight loss exceeding 20% beyond 48 h post-stroke despite all efforts to supplement fluid and diet.
(c) Respiratory distress persisting beyond the first 48 h.
(d) Intermittent abnormal motor activity, suggestive of seizure, tonic clonic seizures, persisting beyond first 72 h, presence of barrel rolling.
(e) No recovery of weight towards the pre-stroke level 7 days post-stroke.

Animals were euthanized using carbon dioxide in a VetTech installation (VetTech Solutions Ltd., Cheshire, UK), following Directive 2010/63/EU of the European Parliament and the European Union Council for Protection of Animals used for scientific purposes. The rats were kept and exposed to all manipulations in compliance with Directive 2010/63/EU of the European Parliament and of the Council. The research was approved by the bioethical commission of the V.M. Gorbatov Federal Research Centre for Food Systems of the Russian Academy of Sciences (protocol #04/2017, dated 22 September 2017).

### 2.3. Neurological Deficit Assessment

To study the rats’ neurological status on Days 0 (prior to surgery), 2, 4, 7, 10, and 13 of the experiment, the McGrow stroke-index scale was used with the modifications of I. V. Gannushkina [34,35] and A. E. Kulchikov [36]. Neurological symptoms according to Gannushkina [34] were recorded in points: animals with mild symptoms were scored up to 2.5 points (lethargy, slowness of movement, weakness of the limbs, tremor, unilateral or bilateral ptosis, and Manege movements), with severe manifestations of neurological disorders scoring from 3 to 10 points (paresis and/or paralysis of the lower extremities, lateral position, and comatose state). Following Kulchikov [36], neurological testing was performed by evaluating the postural reactions, flexion reflex, paw placement reaction, and testing in an open field and pulling up on a crossbar; when testing, involuntary innate behavioral reactions were assigned a score: for values from 1 to 5 points, a mild degree of brain damage was diagnosed, from 6 to 9, it was diagnosed as a medium, and from 10 to 20, it was diagnosed as severe (Table 1). The group average score was calculated from the summary points of each rat.

Motor activity, exploratory behavior, and emotionality were evaluated in the “Open field” test. The studies were carried out on Days 0 (prior to surgery), 2, 3, 6, 7, 10, 11, and 13 of the experiment. The “Open field” installation is a square box (100 × 100 cm) with sides of 45 cm high. The installation arena is divided into 25 squares (20 × 20 cm), necessary for visual registration of the movements of the tested animals. The arena has 16 holes with a diameter of 20 mm at the intersection of the lines of sectors (mink). According to the standard method, the animal was placed in the center of the arena and the behavioral acts were recorded for three minutes: horizontal motor activity––the number of crossed squares; vertical motor activity––the number of racks; the number of examined minks (“mink reflex”); grooming; and the number of acts of defecation (boluses). The arena was cleaned with disinfectant after testing each animal. 

Motor functions were evaluated in the test of holding the animals on a horizontal rod on Days 0 (prior to surgery), 2, 6, 10, and 13 of the experiment. The installation consisted of a horizontal crossbar (2 mm in diameter) placed at a height of 60 cm from the table. The time of holding the animals on the crossbar was recorded, and the animal’s inability to pull its hind legs up to the crossbar was recorded as limb weakness and a manifestation of neurological deficit. Rats’ activity was recorded on a Nikon DT5600 Kit (Nikon, Tokyo, Japan) and automatically processed using RealTimer software (OpenScience, Moscow, Russia).

The experiment was completed on the 15th day, which was associated with a decrease in the severity of the clinical disease in rats on Days 9–14, according to [37]. At the end of the experiment, the rats were euthanized in a euthanasia chamber (VetTech Solutions Ltd., Cheshire, UK) following EU Directive 2010/63/EU. Blood was collected from the stunned animals from the right atrium in EDTA tubes for hematological studies and standard glass tubes for subsequent serum sampling after centrifugation (1300× *g* for 5 min).

The hematological analysis was performed on an Abacus Junior Vet 2.7 automatic analyzer (Diatron Messtechnik GmbH, Wiener Neudorf, Austria) using Diatron reagent kits. Biochemical analysis of the blood serum [38] was performed on a BioChem SA analyzer (HTI, North Attleboro, MA, USA) using reagent kits (HTI, North Attleboro, MA, USA).

To study the morphological parameters of cells, the brain was immediately removed from the cranium and fixed in 10% buffered formalin (HistoSafe, BioVitrum, Sankt-Peterburg, Russia) for 2 hours. The fixed brain was cut coronally through the needle entry site (defined on the surface of the brain) as well as 2 mm in the front and 2 mm behind this plane. Sections of the frontal cerebral cortex 10 microns thick were prepared on a Microm HM 525 cryotome (Carl Zeiss, Oberkochen, Germany). Every 10th section from the rostral to the caudal part of the residual hematoma cavity was stained with hematoxylin–eosin and according to the Nissl method. For the Nissl staining method, sections were defated in xylene (BioVitrum, Sankt-Peterburg, Russia) 2 × 3 min and rehydrated in descending alcohols (100%/95%/70% isopropanol, BioVitrum, Sankt-Peterburg, Russia) for 3 min each. Next, sections were rinsed in distilled water and stained in 0.1% cresyl violet solution (Sigma, Ronkonkoma, NY, USA) for 5 min (warmed up in 37 °C), and then rinsed quickly in distilled water. Then, sections were differentiated in ethyl alcohol (70%/100%/100%, BioVitrum, Sankt-Peterburg, Russia) for 5 min, dehydrated in isopropanol (100%, BioVitrum, Sankt-Peterburg, Russia) for 3 min, cleared in xylene (BioVitrum, Sankt-Peterburg, Russia) 2 × 3 min. Sections were mounted in Bio Mount HM (Bio-Optica, Milan, Italy).

Morphometric studies were performed using a BX 51 microscope (Olympus, Tokyo, Japan). The functional state of the surviving nerve cells was judged based on changes in the area and perimeter of the nuclei and perikaryon of the neurons, the death of neurons and glial cells, and the neuroglial index. Morphometric studies were carried out in a test zone with a size of 270 × 270 μm^2^. Cells with signs of cytolysis and karyolysis, and cells with homogeneously stained acidophilic nuclei, devoid of nucleoli, were considered degenerative [39].

### 2.4. Statistics

Statistical processing of the results was performed in the program STATISTICA version 10.0 (Statsoft, Tulsa, OK, USA). Intergroup comparison was performed using a one-way ANOVA median test and the ANOVA method using the Kruskal–Wallis criterion, with pairwise comparison of groups using the nonparametric Mann–Whitney test. The results are presented as the median (Me) and interquartile range ((P25–P75)). Morphological data are expressed as the mean ± SEM and were analyzed by a two-tailed Student’s *t*-test for unpaired data. A *p* value of 0.05 or less was considered statistically significant.

## 3. Results

### 3.1. Mortality

Within two weeks after the start of the experiment, no deaths of intact animals and sham-operated rats were observed. In the model group of animals, the animals’ mortality amounted to 43.7% for the entire period of the experiment (Table 2); during the 1st day after the operation, 12.5% of the rats with the hemorrhagic stroke model died; further deaths were noted on Days 5, 10, 12, 13, and 14—up to 10% of the animals. 

**Figure 2:**
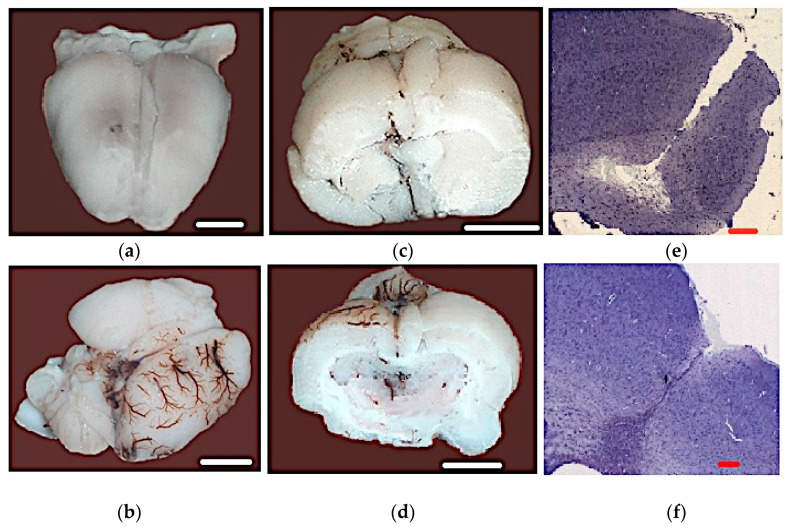
Macro-and microscopic structure of the dead animal brains. (**a**–**d**) Hemorrhages in the structure of the brain; (**e**,**f**) hemorrhage in the capsula interna area: hematoxylin–eosin staining (20×). The lines in the figures represent the scale: white lines—35 mm; red lines—100 microns.

In all cases, mortality was caused by massive hemorrhage into the internal capsule of the brain: the brain was hyperemic, and a subdural hematoma was found on the surface of the right hemisphere of the brain under the dura mater, occupying the parietal and temporal regions (Figure 2) of all animals. A sagittal section revealed massive hemorrhage under the dura mater on the right and left from the lateral and basal regions of both hemispheres. In 12.5% of the dead animals, a thrombus was found under the dura mater (Figure 2c), spreading from the modelling area in the caudal direction to the occipital lobe. Histological studies (Figure 2e,f) revealed the polymorphic nature of the structural and pathomorphological disorders. A significant number of neurons were in a state of acute edema, with swelling of the bodies and their nuclei: neurons with deformed nuclei and signs of their destruction were present. There were dead neurons with signs of autolysis, many diffusely located dead glial cells, pycnosis of the nuclei of some gliocytes, and hyperchromatosis of the vast majority of glial cells.

### 3.2. Weight

When forming groups (Day 0 of the experiment), the Intact animals’ average weight was 247.0 (227.8–261) g. In the Sham and Model rats, the average weights were 218.0 (209.5–220.8) g and 239.0 (229.3–255.3) g and were reduced relative to the values of Group 1 by 11.7% and 3.2%, respectively (*p* < 0.01).

**Figure 3:**
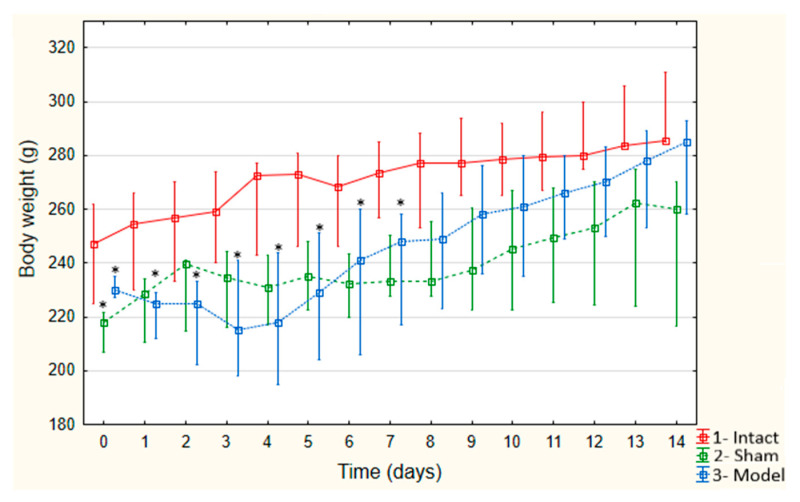
Mass dynamics during the experiment (* *p* < 0.05 in comparison to Group 1—Intact animals).

The mass dynamics in Intact rats throughout the experiment (Figure 3) were favorable; the mass increased by an average of 2.5 ± 0.9 g per day, and the total weight gain by the end of the experiment was 13.5%—285.5 (285.0–304.8).

Sham animals showed a decreased body mass on Day 1 by 10.2% compared to Intact animals; the weight of the animals was 228.5 (211.3–235.5) g (*p* = 0.058). On Day 9, the decrease was up to 14.3% and amounted to 237.5 (225.3–260.3) g (*p* = 0.083); on Day 13, the decrease was up to 7.4% and amounted to 262.5 (228.0–274.5) g (*p* = 0.054). Model animals showed a decrease in weight relative to the Intact animals in the period from 2 to 10 days of the experiment (Figure 3). On Days 2 and 3 of the experiment, the weight decreased by 10.7% and 13.9% (*p* < 0.02), and the weights were 229.5 (209.3–236.0) g and 223.0 (201.0–236.8) g, respectively. Weight loss on Day 4 reached 20.5% (*p* = 0.006), and on Days 5 and 6, weight loss was 16.9% and 15.5% (*p* < 0.02), and the values were 216.5 (196.8–240.3) g, 227.0 (199.0–249.0) g and 220.0 (202.0–256.0) g, respectively. On Day 7, the weight loss reached 20.7%, amounting to 217.0 (204.0–256.0) g (*p* = 0.008). Observations on Days 8–10 revealed a slowdown in weight loss. On Day 8, the weight decreased by 17.0% (*p* = 0.022); on Day 9, the weight decreased 14.8% (*p* = 0.032); and on Day 10, the weight decreased 9.5% (*p* = 0.085)—the values were 230.0 (208.0–259.0) g, 236.0 (199.0–263.0) g, and 252 (208.8–274.0) g, respectively.

On Day 13, the average weight of the Model animals was 285.0 (258.0–293.0) g and did not differ significantly from the values of the Intact (285.5 (285.0–304.7) g) and Sham (260.0 (224.7–269.0) g) animals.

### 3.3. Neurological Assessment

Prior to surgery, there were no absence of neurological deficit symptoms in Intact, Sham, and Model animals.

Results of the animals’ neurological status analysis showed the absence of neurological deficit symptoms in Intact animals during the experiment.

In Sham animals, there were no neurological disorders both on Day 2 after surgery and during follow-up (Table 3).

**Figure 4:**
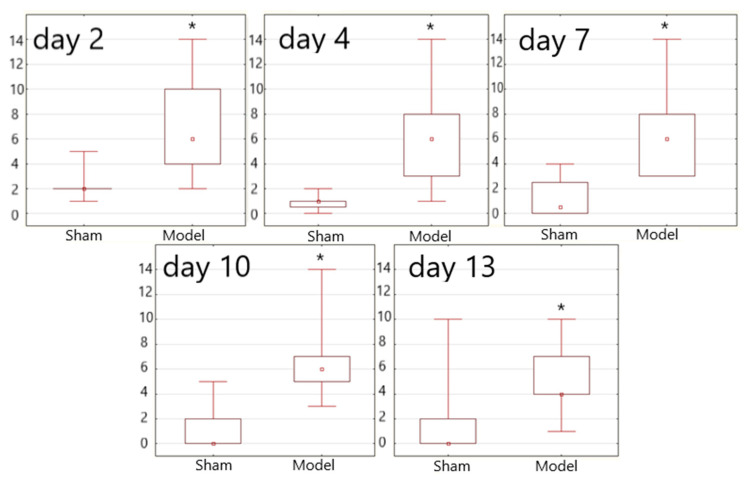
Neurological deficit analysis according to [35]. The data are presented in the form of a swing plot with P25–P75 boundaries and marks of the minimum and maximum values of the dataset. * *p* < 0.05 in comparison to Group 2—Sham animals.

Symptoms such as lethargy, slow motion, and tremors were reported in the Sham animals. The total score, when assessed on the McGrow scale as modified by I.V. Ganushkina, in the group on Day 2 was 0.5 (0.0–0.5), and in A.E. Kulchikov’s modification, it was 2.0 (2.0–2.0) (Figure 4). The neurological deficit in this group was characterized as mild and tended to regress on Day 4. On day 13, 88% of the animals did not have any symptoms of neurological deficit.

In Model animals, from Day 2 after the operation, various neurological symptoms corresponding to a severe deficit were observed, with regression by Day 13 (Table 3). The total scores on Days 2 and 13 on the McGraw scale in I.V. Ganushkina’s modification in the group were 8.5 (4.7–11.6) and 0.7 (0.0–7.7), and according to the scale modified by A.E. Kulchikov, the scores were 6.0 (4.2–9.5) and 5.0 (4.0–6.5) (Figure 4), respectively.

The study of the exploratory behavior in the “Open field” test (Table 4) in Intact rats showed that from Day 7 of the experiment, there was a gradual decrease in the horizontal (from 24.0 (16.8–32.0) to 15.5 (12.8–19.8)) and vertical activity (from 12.0 (8.3–13.5) to 5.0 (2.5–6.8)), exploratory behavior (from 11.0 (6.8–14.5) to 4.0 (1.8–5.5)), and grooming (6.0 (5.3–6.8) to 3.0 (3.0–3.8)), which is associated with the habituation of animals to the experimental setup and their transition to the behavior of ‘patrolling’ familiar territory. However, there were no statistically significant differences relative to Day 0 for the analyzed parameters in animals of this group.

In Sham animals, compared with Intact animals, there was a significant decrease in horizontal activity on Days 2 and 3 of the experiment to 90% (*p* = 0.064; *p* = 0.023, respectively); relative to day 0, this indicator decreased by 88.9% and 86.1%, respectively (*p* < 0.001). Further analysis showed that on Day 7, the horizontal motor activity of the Sham rats was correlated with the intact group and the results of Day 0 (*p* = 1,000). Vertical locomotor activity relative to intact rats decreased on Days 2 and 3 by 12.0 times (*p* = 0.063) and 15.0 times (*p* < 0.001), and on Day 7 by 4.8 times (*p* = 0.014). It was noted that relative to Day 0, the vertical activity of the Sham rats decreased by 5 times (*p* = 0.032) on Day 2 after surgery. The number of hole examinations decreased on Days 2 and 3 by 3.9 (*p* = 0.076) and 9.7 times (*p* = 0.021), respectively. Concerning Day 0, the incidence of hole examination decreased by 83.3% on Day 3 (*p* < 0.05). Grooming in 62.5% of Sham animals was absent on Day 2, and on Day 3, it was absent in 50% of the animals and in general decreased by 13.0 times relative to the Intact animals (*p* = 0.021), by 87.5% relative to Day 0 (*p* = 0.025). The number of grooming acts reached the values of the Intact group on Day 6.

The horizontal activity of the Model animals relative to the Intact ones significantly decreased on Days 2 and 3 by 98.6% (*p* = 0.002) and 87.5% (*p* = 0.019), relative to Day 0, and the activity decreased up to 10 times (*p* < 0.001) on Day 3. From Days 6 to 10, the horizontal motor activity of the Model rats was correlated with the Intact rats (*p* = 1.000). On Day 11, a sharp increase in the activity of the animals was registered, by 3.0 times (*p* = 0.022) compared to the Intact ones. There were no vertical stands on Day 2 of the experiment (*p* < 0.001 relative to the Intact animals and 0 days before the stroke). With further observation on Days 3, 6, and 7, the vertical activity of the animals was still significantly reduced relative to the Intact ones (12.0 times (*p* < 0.001); 4.2 times (*p* = 0.072); 3.0 times (*p* = 0.022), respectively). The number of minks on Days 2 and 3 was significantly less than in the Intact animals (*p* < 0.001); the number of minks was still reduced relative to the Intact ones on Day 6, by 1.75 times (*p* = 0.170), and on Day 7 by 3.7 times (*p* = 0.053). By Day 14, the exploratory activity was comparable to the Intact ones. Grooming was absent in 50.0% of the animals on Day 2; 21.4% had super-intensive grooming. In total, the number of grooming acts decreased 22.0 times (*p* < 0.001) relative to the Intact group. On Day 3, grooming was reduced by 6.5 times (*p* < 0.001); 21.4% of the animals retained super-intensive grooming; and on Days 10 and 11, super-intensive grooming was noted in 38.0%. There were no statistically significant differences relative to Day 0 for grooming in animals of this group (Table 4).

There were no significant differences in the indicators of horizontal motor activity between Sham and Model rats. However, in Model animals relative to Sham rats on Day 2, horizontal motor activity decreased up to 8 times, then on Days 6 and 11 increased by 1.8 and 2.4 times. In the complete absence of vertical activity and mink reflex on Days 2 and 3 in Model animals, horizontal activity in Sham rats was reduced, but not absent. The number of acts of defecation in animals of all groups did not differ significantly. 

In the test of holding on a horizontal rod, the retention time on Day 0 of the experiment was 29.5 (14.5–40.0) s for Intact animals, 30.5 (25.2–39.2) s for Sham rats, and 31.5 (13.2–47.7) s for Model rats, and did not differ significantly.

On Day 2, in Sham animals, the retention time was 15.0 (10.0–20.0) s and was reduced relative to Day 0 by 2.0 times (*p* = 0.455), compared to 20.5 (10.5–35.7), and in Intact animals, it was reduced by 26.8% (*p* = 1.000). On Day 6, the retention time of Sham rats was 18.5 (14.0–30.5) s, and did not differ from the intact values of 19.5 (13.7–29.0) s. On days 10 and 13, the Sham rats were held on the bar for 31.0 (14.2–43.7) s and 26.0 (21.0–36.2) s, and in Intact rats, it was 31.5 (16.5–39.7) s and 15.0 (9.7–27.0) s.

On Day 2, 50% of Model animals showed impaired motor functions; the retention time was 2.0 (0.0–6.7) s and was reduced relative to Day 0 by 15.7 times (*p* < 0.001). The retention time on Day 2 for model rats was also reduced in comparison to Intact rats (Day 2) by 10.2 times (*p* = 0.011); in comparison to the Sham rats (Day 2), by 7.5 times (*p* = 0.027). On Day 6, 35.7% of Model animals showed limb weakness; retention time increased 6.7 times relative to 2 days (*p* = 0.023) and amounted to 13.5 (4.7–50.5) s, which did not differ significantly from Intact and Sham rats. On Day 10, motor dysfunctions were found in 33.3% of rats; the rats hung on the bar for 8.0 (6.0–30.0) s, which was 3.9 times less than the values of Intact and Sham animals (*p* = 0.406 and *p* = 0.198, respectively). On Day 13, motor disorders were observed in 30.0% of the Model animals; the retention time was 8.5 (5.0–15.5) s, which was 3.1 times less than the values of Sham rats (*p* = 0.013).

### 3.4. Results of the Hematological and Biochemistry Analysis

The hematological parameters of the Intact animals were within the physiological norm and corresponded to healthy Wistar rats of the given sex and age (Table 5).

In Sham animals relative to the Intact ones, a decrease in leukocytes content by 18.9% (*p* = 0.793) was revealed, including a decrease in the lymphocytes’ relative content by 7.8% (*p* = 0.362), and a decreased amount of monocytes, eosinophils, basophils, and immature cells by 62.5% (*p* = 0.386) against the background of an increase in the granulocytes’ relative content by 2.3 times (*p* = 0.226).

In Model rats, relative to Intact ones, an increase in the content of monocytes, eosinophils, basophils, and immature cells was noted, by 1.89 times (*p* = 0.002), and the relative content of a mixture of monocytes, eosinophils, basophils, and immature cells increased by 1.4 times (*p* = 0.340). Granulocytes increased by 2.6 times (*p* = 0.126), with a decrease in the relative content of lymphocytes by 13.6% (*p* = 0.089) in Model rats. In addition, an increase in the average volume of erythrocytes by 4.0% (*p* = 0.191) was revealed. Relative to the Sham animals, the Model rats showed an increase in the relative content of a mixture of monocytes, eosinophils, basophils, and immature cells by 3.77 times (*p* < 0.001), granulocytes by 11.0% (p = 0.226), as well as an increase in the average volume of erythrocytes of 6.1 % (*p* = 0.011).

The biochemical parameters of the Intact animals’ serum (Table 6) were within the physiological norm and corresponded to the parameters of healthy Wistar rats of the given sex and age.

In Sham animals relative to the Intact ones, there was an increase in glucose content by 34.7% (*p* = 0.027), creatinine by 22.2% (*p* = 0.027), and decrease in GGT activity by 23.4% (*p* = 0.114).

In Model animals relative to the Intact ones, the albumin content decreased by 8% (*p* = 0.005) and creatinine by 15.6% (*p* = 0.159), with an increase in glucose by 66.4% (*p* = 0.029) and cholesterol by 17, 8% (*p* = 0.044); there was decreased ALT activity by 44.2% (*p* = 0.014). Relative to Sham rats, the ALT activity decreased by 45.1% (*p* = 0.007).

### 3.5. Results of the Histopathological Analysis

**Figure 5:**
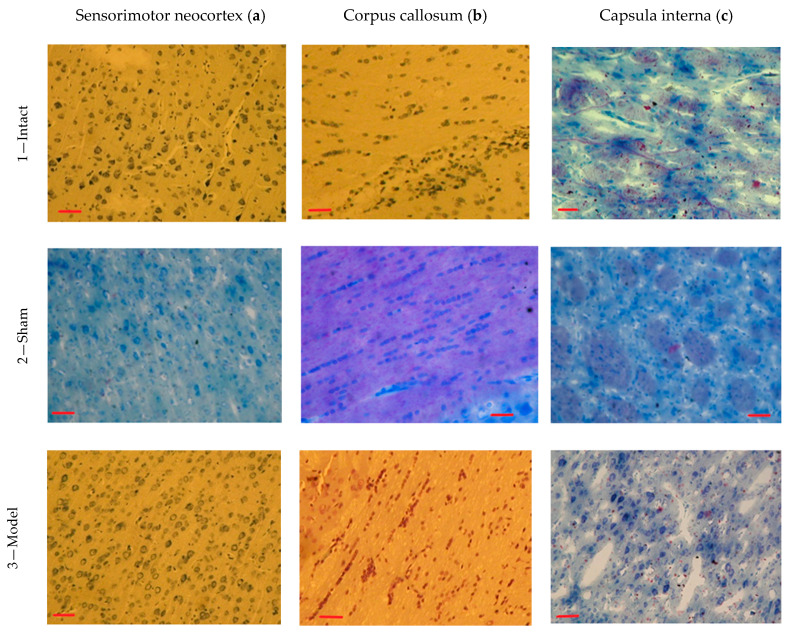
Histological brains structure (200×). Nissl/Hematoxylin–eosin staining. Scale bar 50 µm.

When studying the sensorimotor cortex cerebral hemispheres of the Intact animals, the neocortex structure did not have pronounced signs of pathological process (Figure 5(1a)). Pyramidal neurons were characterized by tigroid background in the cytoplasm, located evenly around the nucleus. Some neurons with a tigroid background were found in dispersed clump form. The neurons’ nuclei occupied a central position in the cells’ cytoplasm. The neurons’ groups with a tigroid background and weak staining of the nuclei were encountered in the neocortex. The shape of the pyramidal neurons was not changed, and the nuclei were large. There were single hyperchromic neurons with shrunken perikarya. Indistinct pericellular edema was observed around such cells. Most often, one nucleolus was present in the nuclei of the pyramidal neurons. No pathological changes in the glial cells and blood vessels were revealed. Signs of perivascular edema were observed around individual blood vessels (Figure 5(1a)).

In the corpus callosum, glial cells were arranged in groups along the main direction of the nerve fibers in a given brain structure. The white matter in this interhemispheric zone of the animals’ brains was basophilic and was not characterized by acidophilic or oxyphilic staining. Glial cell nuclei had a normal shape without signs of pathological changes and processes (Figure 5(1b)). The nuclei of these cells contained single nucleoli. Mild manifestations of perivascular edema were observed around individual blood vessels.

In the capsula interna, diffusely located glial cells were noted (Figure 5(1c)) among the bundles of nerve fibers; however, proliferation phenomena and glial cell hypertrophy were not detected. Some nerve fibers had acidophilic staining against the background of dominant basophilia. There were local perivascular edema and the development of individual perivascular edema in the area of the inner capsule.

In the neocortex of Sham rats, it was found that significant structural changes did not develop in the cerebral vessels; only a slight increase in the perivascular space was noted.

Individual vessels spasm was noted. There were no pathological changes in the vessels and glia; the nuclei of the gliocytes had a normal shape. At the same time, pericellular edema was observed around the glial cells. Pyramidal neurons were characterized by a tigroid background in the cytoplasm located in significant amounts around the nucleus. The nuclei were centrally located in the bodies of the neurons (Figure 5(2a)).

In the corpus callosum, glial cells were arranged in strictly oriented sequences along with groups of nerve fibers. The white matter in this zone had acidophilic staining. The shape of the glial cell’s nuclei was normal, without pathological changes (Figure 5(2b)). In the capsula interna, glial cells were located diffusely (Figure 5(2c)); the proliferation and hypertrophy of glial cells were not established.

In Model animals with a model of left-sided hemorrhagic stroke, in the neocortex, the polymorphic nature of the structural changes was observed. In the stroke hemisphere, most neurons were in a state of acute edema and swelling of the bodies and their nuclei (Figure 5(3a)). Neurons with deformed nuclei and signs of destruction were present. There were dead neurons groups with signs of autolysis. Acute pericellular edema was noted, especially in the area of the apical dendrites. Perivascular edema was also present. Glial cells were also characterized by structural changes. Pycnosis of some cell nuclei was noted. Many dead cells were revealed, which were diffusely located in the cortex. At the same time, hyperchromatosis of the remaining cells was noted (Figure 5(3b)). Pericellular edema around individual cells was clearly visible.

In the corpus callosum, glial cells were located chaotically. There were no signs of active proliferation. Often these cells were kept in small groups. The white matter in this zone had a staining level similar to the previous groups. Glial cell nuclei were small in size with hyperchromic staining (Figure 5(3b)).

In the capsula interna, glial cells were oriented in small groups; around them, there was pericellular edema, and perivascular edema was also noted (Figure 5(3c)).

When analyzing the neurons’ morphometric parameters in the V layer of the sensorimotor neocortex in the cerebral cortex (ipsilateral and contralateral) of Intact rats, it was found that the pyramidal neurons’ area averaged 347.39 ± 6.94 μm, and the area of the nuclei was 159.13 ± 2.65 μm (Table 7). The number of dead neurons in the neocortex sensorimotor area did not exceed 3.0%; the number of dead gliocytes was 2.6%. The neuroglial index averaged 0.82 ± 0.05 units.

In Sham animals, slight changes in the cells and nuclei areas of the giant pyramidal neurons in the V layer of the sensorimotor neocortex were revealed, both in the ipsi and in the contralateral hemisphere. In this case, the pyramidal neurons’ areas were 351.54 ± 5.52 µm² and 337.45 ± 5.21 µm², and the areas of the giant pyramidal neurons’ nuclei were 139.37 ± 2.11 µm² and 121.53 ± 3.41 µm², respectively. The number of dead neurons did not exceed 25.7%; the number of dead gliocytes was 14.2%. The neuroglial index averaged 0.84 ± 0.02 units.

At the same time, in Model animals, the neurons’ area in the V layer of the sensorimotor neocortex in the ipsilateral hemisphere was 447.46 ± 11.45 µm², in the contralateral hemisphere—388.85 ± 9.72 µm²; i.e., it increased sharply relative to the intact values by 28.8% and 10.7% (*p* = 0.089), respectively. The areas of the pyramidal neurons’ nuclei increased, respectively, up to 237.49 ± 5.50 µm² and 171.96 ± 3.59 µm² (by 33.0% and 7.5%). The percentages of dead neurons and gliocytes were 44.9% and 13.4% for the ipsilateral hemisphere and 40.6% and 16.3% for the contralateral hemisphere, respectively. The neuroglial index was 0.92 ± 0.01 units for the ipsilateral hemisphere and 0.96 ± 0.02 units for the contralateral hemisphere.

## 4. Discussion

As a rule, most animal models do not seek to model the entire pathological process, focusing on their individual aspects under the strict control of all other circumstances. At the same time, the goal is to better understand the mechanisms of pathogenesis, as well as to test the effectiveness of various approaches to therapy and prevention. Since none of the models can recreate all aspects of hemorrhagic stroke, it is advisable to use different models when testing the means and methods of therapy. The model with autologous blood injection stimulates the development of stroke by the hematoma mechanism. The advantage of these models is the ability to control the volume of blood injected, which contributes to good reproducibility. In addition, in such models, the volume of blood injected correlates with the degree of brain tissue damage and the severity of the functional disorders [6]. 

However, when analyzing the data and interpreting the results obtained in these models, it is necessary to take into account that they reproduce a pathology with a constant hematoma volume, while in the clinic, a secondary increase in the hematoma is possible. In the present study, we characterized the development of disorders in hemorrhagic damage to the tissues of the inner capsule of the left hemisphere of the rat brain when administered autologous blood by the method [20]. 

In the model we studied, the death of animals was observed during the 14-day experiment; the total mortality of the model was 43.7%. The critical days were Days 1, 5, and 12–14; it is expected that during this period, the probability of death increases when this model is replicated. In people with hemorrhagic stroke, the mortality rate during the first month is more than 30% [6,40].

In deceased animals in the capsula interna region of the ipsilateral hemisphere, signs of acute pericellular edema were detected, especially in the apical dendrites; also, a significant number of dead neurons with signs of autolysis; acute edema of neurons with swelling of their nuclei; a lot of diffusely located dead glial cells with hyperchromatosis of the vast majority of glial cells; and, in some glyocytes, pycnosis of the nuclei. So, the model causes neuronal death and is accompanied by pronounced gliosis and glial cell proliferation due to the development of a local inflammatory process, which was also noted [41,42] and can be used for verification methods of treating brain edema and secondary inflammation after intracerebral hemorrhage [37,43]. Thus, an inflammatory response occurs early in patients with cerebral hemorrhage and influences and predicts the disease course [44,45].

Various neurological disorders were observed in the surviving animals throughout the entire period of the experiment. On Day 2 after the operation, a comatose state was registered in 28% of the animals (they subsequently died); the remaining animals were found to have paralysis and paresis of the contralateral limbs, weakness and insensitivity of the limbs, clockwise Manege movements, unilateral and bilateral ptosis, and exophthalmos. In animals, severe motor disorders were registered, manifested in the inability to hold on to a horizontal rod, which persisted up to 13 days in 30% of rats; at the same time, the retention time on the rod was significantly reduced. Neurological deficit led to inhibition of the periods of adaptation and development of research behavior. It is important to note that the increase in horizontal activity from Day 6 to day 11 was due to the Manege movements of rats and is imaginary. During observations, there were manifestations of both over-intensive grooming (on Days 2 and 11 in 21% and 38% of rats, respectively) and its absence (in 50% and 30% of animals, respectively). Violations persisted until the end of the study; 10% of the rats remained in a serious condition, 50% of the animals had an average degree of neurological deficit, and 40% of the animals had mild symptoms of neurological manifestations. The observed neurological disorders correspond to the characteristic symptoms of stroke patients, and their safety correlates quite well with 70–75% of disability in survivors of hemorrhagic stroke [46].

Brain section histomorphometric studies of the animals in the stroke model revealed a significant number for both neurons (up to 45%) and glia (up to 14%); in the ipsilateral hemisphere, most neurons were in a state of acute edema, with swelling of bodies and their nuclei; significant numbers of neurons with deformed nuclei and signs of destruction were detected. There was acute recellular edema in the apical dendrites, glial cells with structural changes, and pycnosis of the nuclei, also described in [47]. Such inflammatory processes led to a shift in the leukocyte formula in the direction of increasing the relative content of granulocytes and a mixture of monocytes, eosinophils, basophils, and immature cells, with a decrease in the relative content of lymphocytes. In addition, there was an increase in the average volume of red blood cells, which may be a delayed response to acute hematoma in stroke modelling. The increase in glucose and cholesterols is associated with the mechanism of compensation for the increased metabolic needs of the body as a whole, and especially the brain. A decrease in serum albumin, creatinine, as well as ALT activity, are predictors of functional outcomes acute stroke [48,49] and confirms the validity of the model.

Post-stroke weight loss of rats increased up to 10 days after the simulation, and by the end of the experiment, it corresponded to the weight of the Intact animals. Weight loss is associated with the development of acute cerebral circulatory disorders and brain edema, which affects the feeding behavior of animals due to compression of the anterior hypothalamus. Thus, body weight is an indirect indicator of hemorrhagic brain damage, which is also confirmed by other studies [50].

For comparison, a group of Sham-operated animals was introduced into the experiment, which underwent an identical craniotomy without damage to the brain structures. No deaths of rats were recorded. In Sham-operated animals, the weight loss on Day 1 after surgery was caused by the consequences of the anesthesia and craniotomy, which also led to the appearance of symptoms of mild neurological deficit. On Days 2 and 3 after trepanation, vertical and horizontal activity, research behavior, and grooming of rats, as well as retention time on the horizontal bar, were significantly reduced. By the end of the experiment, 12.5% of the rats showed lethargy of movement, tremor, weakness of the limbs, and unilateral partial ptosis, as well as weight loss. In other animals, all the analyzed parameters corresponded to the values of the Intact animals. Histological studies of the brain of Sham-operated rats showed no pathological changes in blood vessels, neurons, and glia. However, blood analyses revealed minor changes in the leucocyte count (decrease in the relative content of lymphocytes of a mixture of monocytes, eosinophils, basophils, and immature cells and an increase in the relative content of granulocytes), minor hyperglycemia, and an increase in the creatinine level and GGT activity. It can point to kidney injury after anesthesia [51] or inflammatory processes at the site of injury since the animals were not kept in sterile conditions.

It is worth noting that if it is necessary to conduct molecular studies of the brain, blood sampling from the heart should be carried out in the animal under anesthesia (for example, Zoletil/Xyla) after the rib cage dissection, after taking blood from the right atrium, with the animal immediately transcardially perfused with heparinized saline (0.9%) followed by a periodate lysine paraformaldehyde solution [52], to dissect and isolate the needed brain structures.

## 5. Conclusions

Reproduction of an acute intracerebral hematoma in the left hemisphere of the rat brain according to the method in [20] causes an acute functional deficit, which is manifested by a pronounced violation of motor functions, paralysis, paresis, weakness and insensitivity of the limbs, Manege movements, ptosis, and exophthalmos. These symptoms persist for at least 14 days. In parallel, there was marked edema, neuronal death, and gliosis. The data obtained prove that this model fully reflects the observed clinical situation and reproduces the main diagnostic criteria for acute cerebral circulatory disorders. 

It is known that up to 85% of hemorrhagic strokes develop by the mechanism of hematoma and only about 15% by the mechanism of diapedesis [45]. In this regard, the model with the introduction of autologous blood, which is the method of [20], is in many aspects more relevant. The advantages of these models are good reproducibility of the size and position of the hematoma, preservation of near-hematomic brain tissue, and the ability to change the severity of the structural damage and neurological consequences [26].

A comprehensive study of the local intracerebral hematoma modelling performed in this paper [18] will allow not only to better understand the pathophysiology of the acute period in this model but also to improve the quality of in vivo studies aimed at correcting acute cerebral circulatory disorders.

## Figures and Tables

**Table 1:** Neurological deficit in the animals after assessment, using the McGrow stroke-index scale with A. E. Kulchikov’s modification [36].

Manipulations	Neurological Status Assessment in Points
The animal was brought to the table, the back of the front legs should touch the edge of the table.	Normally, the animal puts both paws on the table, if the animal does not place the paralyzed limb on the table—1 point; does not put both limbs—2 points.
The animal was fixed by the tail above the table and gradually lowered so that the vibrissa touched the table.	Normally, the animal tries to grab the edge of the table with its front paws, if there is no reaction, 2 points are assigned.
The animal was fixed by the tail above the table and gradually lowered so that the vibrissa did not touch the edge of the table.	Normally, the animal tries to grab onto the edge of the table with its front paws, if there is no reaction, 2 points are assigned.
The animal was tested under standard “Open field” conditions.	If the animal makes circular movements in the direction of the paralyzed limb around itself, without going beyond the square—1 point; if circular movements in the direction opposite to the paralyzed limb—2 points
	If the animal makes a circular motion within the central squares, then it is assigned 2 points.
Examination of the eyeballs.	If, after modeling the pathology, ptosis of one eye occurs—1 point; if ptosis of both eyes—2 points; if exophthalmos develops—2 points.
The animal was fixed by the tail and gradually raised.	If the hind legs are crossed—2 points.
Holding on a horizontal rod.	Normally, the animal pulls itself up on the crossbar, if the animal does not pull up and falls—2 points.
Prick in the paw.	If the animal withdraws its paw with a defensive reaction—0 points; if there is no defensive reaction and there is a withdrawal—1 point; if both reactions are absent—2 points.

**Table 2:** Animal mortality during the experiment.

Animal Group	Day 1	Day 5	Day 10	Day 12	Day 13	Day 14	Total Mortality
1—Intact	0/6	0/6	0/6	0/6	0/6	0/6	0/6
2—Sham	0/8	0/8	0/8	0/8	0/8	0/8	0/8
3—Model	2/16	1/14	1/13	1/12	1/11	1/10	7/16

**Table 3:** Results of the assessment of neurological deficit according to [26]. In the table, colored cells give the percentage of animals with recorded symptoms. Group average score presented as Me, (P25–P75).

Symptoms	Points	Day 2		Day 4		Day 7		Day 10		Day 13	
		Sham	Model	Sham	Model	Sham	Model	Sham	Model	Sham	Model
Lethargy, slowness of movement	0.5	62	71	50	64	37	31	25	33	12	40
Tremor	1.0	12	28	12	14	12	15	12	17	12	10
Unilateral partial ptosis	1.0	0	50	0	50	0	38	0	33	0	20
Bilateral partial ptosis	1.5	12	0	12	0	12	8	12	8	12	0
Inability to pull back a limb while holding it	1.5	0	71	0	71	0	69	0	33	0	20
Unilateral ptosis	1.5	0	21	0	36	0	23	0	17	0	10
Bilateral ptosis	1.5	0	28	0	14	0	15	0	8	0	10
Manage movements	2.0	0	57	0	50	0	38	0	17	0	10
1 limb paresis	2.0	0	0	0	0	0	0	0	0	0	0
2 limb paresis	3.0	0	43	0	57	0	46	0	17	0	10
1 limb paralysis	3.0	0	28	0	14	0	15	0	8	0	10
2 limb paralysis	4.0	0	0	0	0	0	0	0	0	0	0
Coma	7.0	0	28	0	14	0	15	0	17	0	10
Fatal outcome	10.0	0	12	0	0	0	7	0	8	0	17
Group average score	Me	0.5	8.5	0.2	6.0	0.0	5.0	0.0	1.0	0.0	0.7
	P25	0.0	4.7	0.0	1.0	0.0	1.0	0.0	0.0	0.0	0.0
	P75	0.5	11.6	0.5	8.5	0.1	8.7	0.0	9.5	0.0	7.7

**Table 4:** Motor activity and orientation–exploratory behavior of rats in the “Open field” test. The results are presented as Me, (P_25_–P_75_). In the table, colored cells allow to focus on the most important parameters.

Groups	Day 0	Day 2	Day 3	Day 6	Day 7	Day 10	Day 11	Day 13
**Horizontal Activity, Number of Crossed Squares**								
1—Intact	45.5 (32.8–55.3)	35.0 (19.3–54.5)	44.0 (32.8–54.5)	20.5 (9.8–50.8)	24.0 (16.8–32.0)	19.0 (8.5–36.3)	12.5 (5.8–17.8)	15.5 (12.8–19.8)
2—Sham	36.0 (31.3–39.5)	4.0 (2.0–9.3)	5.0 * (2.8–7.0)	9.5 (4.8–23.5)	15.0 (10.3–29.5)	16.5 (12.0–19.5)	16.5 (9.3–26.5)	37.0 (11.3–43.3)
3—Model	50.5 (32.3–53.5)	0.5 * (0.0–4.0)	5.5 * (0.0–23.0)	17.0 (6.3 –40.8)	17.0 (6.0–33.0)	24.0 (12.0–54.0)	40.0 * (26.0–43.0)	22.0 (10.3–45.3)
**Vertical Activity, Number of Racks.**								
1—Intact	11.0 (5.8–12.5)	12.0 (7.0–17.0)	15.0 (10.8–17.8)	12.5 (6.0–21.3)	12.0 (8.3–13.5)	7.5 (5.3–11.3)	6.5 (4.5–7.0)	5.0 (2.5–6.8)
2—Sham	5.0 (2.8–9.3)	1.0 (0.8–1.0)	1.0 * (0.8–1.3)	2.5 (1.0–7.0)	2.5 * (1.8–4.0)	3.0 (1.5–6.0)	3.0 (1.0–4.3)	1.0 (0.0–3.3)
3—Model	12.5 (6.3–16.5)	0.0 * (0.0–0.0)	1.0 * (0.0–1.0)	4.0 (1.0–6.0)	4.0 * (1.0–7.0)	3.0 (1.0–10.0)	5.0 (3.0–11.0)	3.5 (0.8–6.5)
**Mink Reflex, Number of Examined Minks**								
1—Intact	11.0 (8.8–12.5)	13.5 (12.3–17.0)	14.5 (13.3–15.8)	7.0 (5.0–12.8)	11.0 (6.8–14.5)	6.5 (4.0–11.3)	5.0 (4.3–5.0)	4.0 (1.8–5.5)
2—Sham	9.5 (7.5–11.3)	3.5 (1.0–5.5)	1.5 * (1.0–2.3)	3.0 (1.8–6.0)	2.5 (1.0–6.8)	3.0 (0.8–4.3)	2.5 (0.0–4.3)	4.5 (0.8–9.0)
3—Model	10.0 (8.5–18.0)	0.0 * (0.0–2.0)	0.0 * (0.0–2.0)	4.0 (1.3–6.8)	3.0 * (1.0–7.0)	4.0 (1.0–7.0)	3.0 (1.0–6.0)	3.0 (0.8–6.0)
**Grooming, Number of Acts**								
1—Intact	5.0 (3.3–6.0)	11.0 (6.5–12.5)	6.5 (6.0–7.0)	5.0 (2.5–6.8)	6.0 (5.3–6.8)	6.5 (4.5–7.8)	3.5 (3.0–4.0)	3.0 (3.0–3.8)
2—Sham	4.0 (3.0–5.0)	0.0 * (0.0–1.0)	0.5 * (0.0–1.0)	4.0 (2.8–4.3)	4.0 (3.0–4.3)	3.5 (1.8–5.5)	3.5 (1.8–5.3)	2.0 (0.0–4.0)
3—Model	4.0 (2.0–4.8)	0.5 * (0.0–4.8)	1.0 * (0.0–4.0)	4.0 (0.0 –5.8)	4.0 (2.0–5.0)	1.0 (0.0–8.0)	1.0 (1.0–9.0)	3.0 (0.8–4.3)

* *p* < 0.05 in comparison to Group 1—Intact animals.

**Table 5:** Results of the animals’ blood hematological analysis at the end of the experiment. The results are presented as Me, (P_25_–P_75_). In the table, colored cells allow to focus on the most important parameters.

Parameters	Group of Animals		
	1—Intact	2—Sham	3—Model
LEU, 10^9^/L	7.45 (6.16–9.70)	6.04 (5.22–7.50)	7.90 (5.72–8.25)
LYM, 10^9^/L	7.00 (5.54–8.22)	5.01 (4.47–6.03)	6.02 (5.22–6.44)
Relative LYM, %	91.25 (87.95–92.85)	84.10 (77.95–87.78)	78.80 (74.40–86.35)
MID, 10^9^/L	0.09 (0.08–0.13)	0.04 (0.03–0.05)	0.17 ^#^ (0.15–0.31)
Relative MID, %	1.60 (0.90–2.00)	0.60 (0.60–0.70)	2.25 ^#^ (1.93–3.98)
GRAN, 10^9^/L	0.57 (0.33–1.03)	0.95 (0.76–1.17)	1.19 (0.56–1.86)
Relative GRAN, %	6.55 (5.55–10.55)	15.30 (10.30–21.90)	17.00 (8.53–23.83)
RBC, 10^12^/L	7.69 (7.65–8.17)	7.82 (7.67–8.00)	7.43 (7.25–8.00)
HGB, g/L	130.50 (125.50–136.50)	129.50 (127.25–144.25)	131.00 (127.25–137.75)
HCT, %	39.79 (38.16–41.15)	39.16 (38.11–39.61)	39.08 (38.32–39.95)
MCV, mkm^3^	50.00 (49.00–51.00)	49.00 (48.75–50.25)	52.00 ^#^ (51.00–52.00)
PLT, 10^9^/L	733.50 (709.00–809.50)	751.50 (660.75–826.00)	759.00 (719.00–829.25)
PCT, %	0.51 (0.47–0.56)	0.48 (0.43–0.54)	0.50 (0.48–0.55)

^#^—*p* < 0.05 in comparison to 2—Sham animals; Abbreviations: LEU—leukocytes; LYM—lymphocytes; GRAN—granulocytes; MID—a mixture of monocytes, eosinophils, basophils and immature cells; RBC—red blood cells; HGB—hemoglobin; HCT—hematocrit; PLT—platelets; PCT—plateletcrit; MCV—average erythrocyte volume.

**Table 6:** Results of animal blood serum biochemical analysis at the end of the experiment. The results are presented as Me, (P_25_–P_75_). In the table, colored cells allow to focus on the most important parameters.

Parameters	Group of Animals		
	1—Intact	2—Sham	3—Model
Total protein, g/L	65.30 (64.65–66.90)	63.15 (62.20–65.25)	68.10 (66.30–68.75)
Albumin, g/L	45.20 (43.70–45.60)	43.25 (42.38–43.95)	41.60 * (41.40–41.85)
Glucose, mmol/L	10.80 (9.10–13.58)	16.55 * (16.00–17.60)	17.97 ^#^ (14.05–19.3)
Bilirubin indirect, μmol/L	3.20 (2.90–3.40)	3.28 (3.15–4.25)	3.50 (3.10–3.80)
Bilirubin direct, μmol/L	1.80 (1.65–1.90)	1.75 (1.68–1.95)	1.90 (1.85–2.15)
Creatinine, μmol/L	54.50 (51.75–56.50)	42.38 * (42.00–47.33)	46.00 (44.75–48.25)
Urea, mmol/L	6.15 (5.62–6.57)	6.60 (5.53–7.56)	6.07 (5.91–6.64)
AST, E/L	132.65 (130.17–133.97)	128.34 (117.39–149.97)	136.20 (115.24–143.12)
ALT, E/L	31.00 (26.50–34.00)	31.50 (28.00–33.18)	17.30 *^, #^ (16.00–25.00)
ALP, E/L	175.50 (158.40–199.50)	146.05 (131.05–173.55)	166.90 (154.85–177.40)
GGT, E/L	2.65 (2.52–2.84)	2.03 (1.87–2.26)	2.43 (2.23–2.70)
LDH, E/L	404.56 (383.39–461.42)	521.34 (456.84–624.32)	556.00 (328.13–595.01)
Cholesterol, mmol/L	1.57 (1.47–1.67)	1.79 (1.71–1.92)	1.85 * (1.70–2.21)
Triglycerides, mmol/L	1.20 (0.85–1.55)	1.25 (1.08–1.41)	1.40 (1.34–1.55)

* *p* < 0.05 in comparison to 1—Intact animals; ^#^ *p* < 0.05 in comparison to 2—Sham animals. Abbreviations: GGT—gamma-glutamyl transpeptidase; ALT—alanine aminotransferase; LDH—lactate dehydrogenase; AST—aspartate aminotransferase; ALP—alkaline phosphatase.

**Table 7:** The neurons’ morphometric parameters in the V layer of the sensorimotor neocortex.

Parameters		Group of Animals				
		1—Intact	2—Sham		3—Model	
			Ipsi	Contr	Ipsi	Contr
Area of the pyramidal neurons, μm	Mean	347.39	351.54	337.45	447.46 *	388.85
	SEM	6.94	5.52	5.21	11.45	9.72
Area of the nuclei, μm	Mean	159.13	139.37	121.53	237.49 *	171.95
	SEM	2.65	2.11	3.41	5.50	3.59
Neuroglial index	Mean	0.82	0.85	0.83	0.92	0.96
	SEM	0.05	0.02	0.02	0.01	0.02

* *p* < 0.05 in comparison to Group 1—Intact animals.

## Data Availability

Not applicable.
